# Experimental Yellow Fever in the Squirrel Monkey (*Saimiri* spp.): Hematological, Biochemical, and Immunological Findings

**DOI:** 10.3390/v15030613

**Published:** 2023-02-23

**Authors:** Milene S. Ferreira, Lívia C. Martins, Karla F. L. de Melo, Wellington B. da Silva, Aline A. Imbeloni, José Augusto P. C. Muniz, Camille F. de Oliveira, Maria Nazaré O. Freitas, Éder B. dos Santos, Liliane L. Chagas, Márcia B. M. Luz, Luiz A. D. de Queiroz, Robert B. Tesh, Pedro F. C. Vasconcelos

**Affiliations:** 1 Department of Arbovirology and Hemorrhagic Fevers, Evandro Chagas Institute, Ananindeua 67030000, PA, Brazil; 2 Postgraduate Program in Biology of Infectious and Parasitic Agents, Federal University of Pará, Belém 66075110, PA, Brazil; 3 National Primate Center, Evandro Chagas Institute, Ananindeua 67030000, PA, Brazil; 4 Department of Pathology, University of Texas Medical Branch, Galveston, TX 77555, USA; 5 Department of Pathology, Pará State University, Belém 66050540, PA, Brazil

**Keywords:** experimental infection, yellow fever, animal models, pathogenesis, *Saimiri* spp.

## Abstract

Between 2016 and 2018, Brazil experienced the largest sylvatic epidemic of yellow fever virus (YFV). Despite to the magnitude and rapid spread of the epidemic, little is known about YFV dispersion. The study evaluated whether the squirrel monkey is a good model for yellow fever (YF) studies. Methods: Ten animals were infected with 1 × 10^6^ PFU/mL of YFV, with one negative control. Blood samples were collected daily during the first 7 days and at 10, 20 and 30 days post infection (dpi) for detection of viral load and cytokines by RT-qPCR; measurements of AST, ALT, urea and creatinine were taken; IgM/IgG antibodies were detected by ELISA, and hemagglutination inhibition and neutralization tests were performed. The animals exhibited fever, flushed appearance, vomiting and petechiae, and one animal died. Viremia was detected between 1 and 10 dpi, and IgM/IgG antibodies appeared between 4 and 30 dpi. The levels of AST, ALT and urea increased. The immune responses were characterized by expression of S100 and CD11b cells; endothelial markers (VCAM-1, ICAM-1 and VLA-4), cell death and stress (Lysozyme and iNOS); and pro-inflammatory cytokines (IL-8, TNF-α, and IFN-γ) and anti-inflammatory cytokines (IL-10 and TGF-β). The squirrel monkeys showed changes similar to those described in humans with YF, and are a good experimental model for the study of YF.

## 1. Introduction

The changing epidemiological profile of yellow fever virus (YFV) (*Flaviviridae-Flavivirus*) in Brazil has resulted in human cases being reported in more than 10 states, with the Southeast region being the most affected in the last three years. This unexpected emergence in the Southeast region raises the need for new studies to better understand the factors related to the dispersion and re-emergence of YFV [1].

The intense population growth and movement of people into previously forested areas, as well as the exploitation of natural resources in forested areas, has resulted in the destruction of the natural ecosystems where non-human primates (NHPs) live and has forced the animals to migrate and seek new environments and to colonize forest areas near urban centers, where the potential vectors of urban YF might be abundant [2]. Lima et al. [3] demonstrated the vector competence of *Ae. aegypti* and *Ae. albopictus* for different genetic lineages of YFV (South American and African genotypes). The recent detection of the YFV genome in *Ae. albopictus* in Brazil is of concern and raise the possibility of the establishment of an intermediate transmission cycle in peri-urban or urban areas vectored by the Asian tiger *Ae. albopictus* (L C Martins, Material not intended for publication. Evandro Chagas Institute, Ananindeua, Pará).

This points to the outstanding role of NHPs as primary YFV hosts, viral amplifiers, and sources of information on the dynamics of the virus circulation during and between epidemics. Considering the above, we decided to evaluate aspects of YFV interaction and immunological response in the squirrel monkey (*Saimiri* spp.), an NHP commonly found in Brazilian urban centers, and particularly abundant in the Amazon region.

## 2. Materials and Methods

### 2.1. Virus

A South American genotype I isolate of YFV (BeH655417), originally obtained from a non-fatal human case at the Department of Arbovirology and Hemorrhagic Fever of the Evandro Chagas Institute (SAARB/IEC) Ananindeua, Pará, Brazil, was used to infect the animals. For the production of viral stock, a single additional passage was carried out in a continuous mosquito cell lineage of *Ae. albopictus* (clone C6/36) [4], and confirmation of infection by YFV was carried out by indirect immunofluorescence using monoclonal antibodies (Bio Manguinhos—FIOCRUZ/MS/SVS) [5].

### 2.2. Experimental Infection

Eleven squirrel monkeys (*Saimiri* spp.)—males, adults (6 to 7 years old) with an average weight of 786 g from a breeding colony at National Primate Center (CENP) Ananindeua, Pará, Brazil—were used in this study. All animals were negative for anti-flavivirus antibodies (YFV, *dengue Virus* 1–4, *Ilheus Virus*—ILHV, *Rocio Virus*—ROCV, *Sant Louis encephalitis Virus*—SLEV) by hemagglutination inhibition test (HI) [6,7].

Prior to infection, the animals were weighed (Micheletti-Mic Baby scale, São Paulo, Brazil) and bled from the femoral vein for general blood tests. Subsequently, ten animals were infected intradermally with 0.5 mL (infectious dose: 1 × 10^6^ plaque forming units—PFU/mL) of C6/36 cell culture supernatant of BeH655417 YFV isolate. One animal served as a noninfected control until the end of experimental kinetics. The infected animals were observed every 2 h for a period of 30 days to evaluate any behavioral and clinical changes, according to the scan sampling method [8]. Body weight, temperature, body score, and hydration were measured daily. All procedures were performed at biosafety level 3 at the IEC.

### 2.3. Collection of Biological Samples

During the first seven days and on the 10th, 20th and 30th days post infection (dpi), one infected animal with YFV was immobilized and anesthetized by intramuscular route with Zoletil (50 mg/mL, dose 5.5 mg/kg) [9]. Ten mL of blood was obtained by femoral vein.

### 2.4. YFV Viral Load and Cytokines

RNA extraction was carried out using the Maxwell 16 Viral Total Nucleic Acid Purification commercial kit (Promega, Madison, WI, USA) and the Maxwell extraction platform (Promega). The concentration and purity of the RNA were determined by Qubit 2.0 (Invitrogen Life Technologies, Carlsbad, CA, USA) with the Qubit RNA BR assay kit (Invitrogen Life Technologies) and extracted RNA quality was evaluated by 2100 Bioanalyzer Instrument (Agilent Technologies, San Diego, CA, USA) using the Agilent RNA 6000 Nano commercial kit (Agilent Technologies).

Quantification of viral load was carried out by the method described by Domingo et al. [10], using the commercial GoTaq Probe 1-Step RT-qPCR System kit (Promega), together with the absolute quantification method plasmid cloned into vector pGEM Easy (Promega) from the YFV genome. For the Real-time quantitative PCR (RT-qPCR) the GoTaq qPCR Master Mix kit (Promega) and the ABI Prism 7500 Sequence detection system (Applied Biosystems, Foster City, CA, USA) were used. The primers (Supplemental material Table S1) used for the target cytokines were obtained using the Primer 3 program [11].

The genes of β-actin and GAPDH 18S were used as endogenous controls. Relative quantification of mRNA expression was calculated by comparing the Ct value (Threshold cycle) which is determined by the RQ equation 1/4 2^−ΔΔCT^ [12]. Relative quantification values were scattered on a Gaussian curve and Student’s t test was used to verify mRNAs which had differentiated expression between the infected samples and the negative control samples.

### 2.5. Serology

The capture of anti-YFV IgM antibodies by ELISA was performed as described by Martin et al. [13], and optical density (OD) was expressed with a 450 nm filter. To detect total antibodies against YFV (wild and vaccinal strain—17D), DENV 1-4, ILHV, SLEV, ROCV, the HI test was performed [6,7]. Positive results were expressed as titers from 1:20 to ≥1:280.

### 2.6. Hemogram and Coagulogram

The hemogram was performed on blood collected with EDTA before and after infection. For this, the Cell—Dyn Ruby (Abbott^®^, Chicago, IL, USA) apparatus was used according to recommendations of the manufacturer. The following parameters were measured: total red blood cells, hemoglobin, hematocrit, leukocytes (basophils, neutrophils, monocytes and lymphocytes) and platelets. To perform the coagulogram, plasma was obtained with sodium citrate in the CLOT timer-laser sensor (Sorocaba, São Paulo, Brazil) device according to the manufacturer instructions. In the coagulogram, prothrombin time (PT) and activated partial thromboplastin time (APTT) were measured.

### 2.7. Biochemistry

Total blood urea and creatinine were measured using commercial kits (ROCHE, Munich, Germany) and the biochemical analyzer Architect C4000 Abbott^®^. Measures of aspartate aminotransferase (AST) and alanine aminotransferase (ALT) were performed using a Vitros^®^ Johnson and Johnson analyzer, New York, NY, USA. Results are expressed as UL/mL.

### 2.8. Data Analysis

The variables observed were quantified and presented as graphs of lines and bars using the Graph Pad Prism 3.0 program for Windows (GraphPad software, San Diego, CA, USA). The data from hemogram, coagulogram and biochemistry were transformed into a ratio where values were considered before and after infection. All values ranging from 0.75 to 1.5 (gray range) were considered to have changed. In other words, values of parameters <0.75 were considered below normal and values >1.5 were considered above normal [14]. The results obtained in the HI test were analyzed on the basis of a score from 1 to 6 where 1 = 1:20, 2 = 1:40, 3 = 1:80, 4 = 1:160, 5 = 1:320, 6 = ≥1:640. For the analysis of correlation between the viral load and the expression of cytokines, the Wilcoxon test was used and results with *p* < 0.05 was considered statistically significant.

## 3. Results

Animals were observed daily for a period of up 30 days. At the beginning of the experiment, the animals showed restlessness (1–2 dpi), probably due to handling and manipulation. At 5 and 6 dpi, the animals appeared clinically ill. At 6 dpi, one animal evolved to death. Most animals manifested increased heart rate (1, 3 and 11 dpi), respiratory rates (1–12 dpi), dehydration (5–7 dpi), fever ranging from 100.58 °F (38.1 °C) to 105.08 °F (40.6 °C) (3–6 dpi), redness (1–3 dpi), chills (2–5 dpi), vomiting and diarrhea (4 dpi), and petechiae (6–10 dpi), and were apathetic and slightly prostrate on days 4–9 dpi.

**Figure 1:**
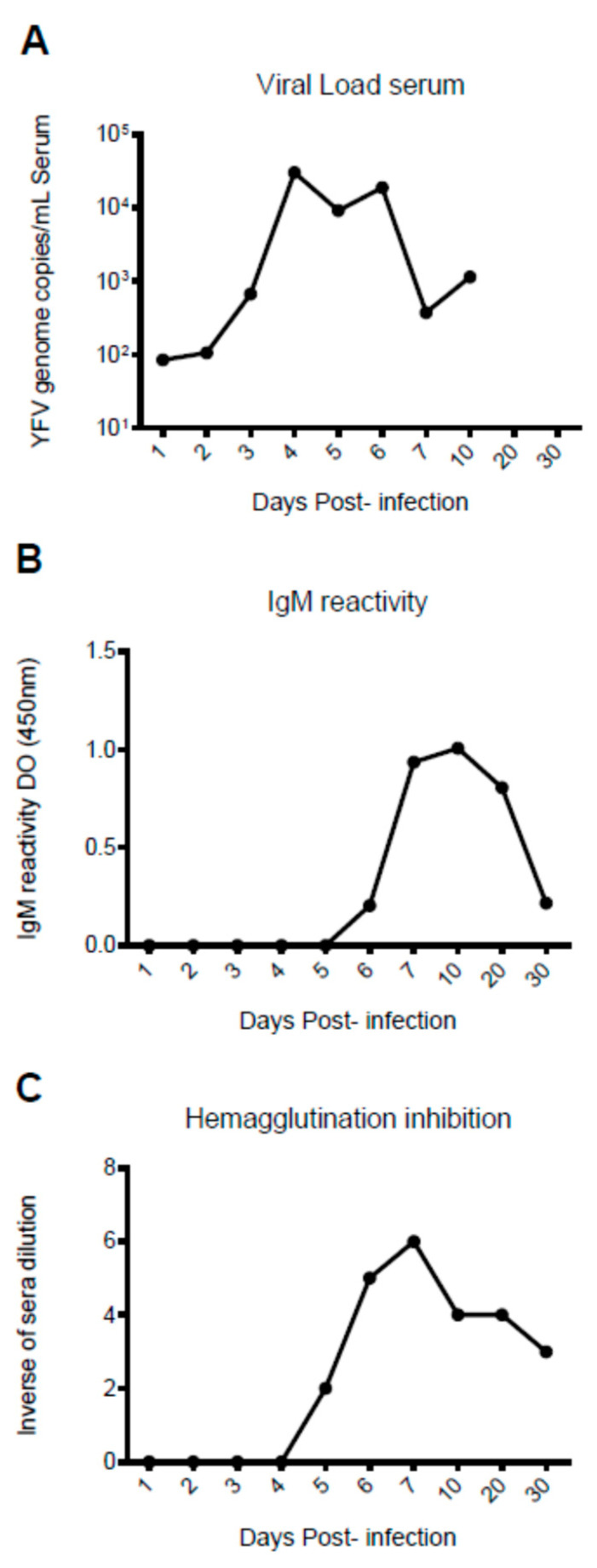
Detection of viral genome and anti-YFV antibodies in squirrel monkeys (*Saimiri* spp.) infected with yellow fever virus. (**A**) Detection of the viral genome by real-time RT-PCR; (**B**) detection of anti-YFV IgM antibodies by enzyme immune assay; (**C**) detection of total antibodies by HI technique.

YF viral genome was detected from 1 to 10 dpi, with peaks at 4 dpi (30,550 copies/μL) and 6 dpi (18,989 copies/μL) (Figure 1A). Anti-YFV IgM antibodies were detected by ELISA and total antibodies by HI test from the 5 dpi until the end of the experiment (30 dpi) (Figure 1B,C). The HI test results also revealed some cross-reactivity between YFV (wild and vaccinal strain—17D) and other flaviviruses (DENV 1-4, ILHV, SLEV and ROCV) up until 30 dpi (Supplementary Materials, Table S2).

**Figure 2:**
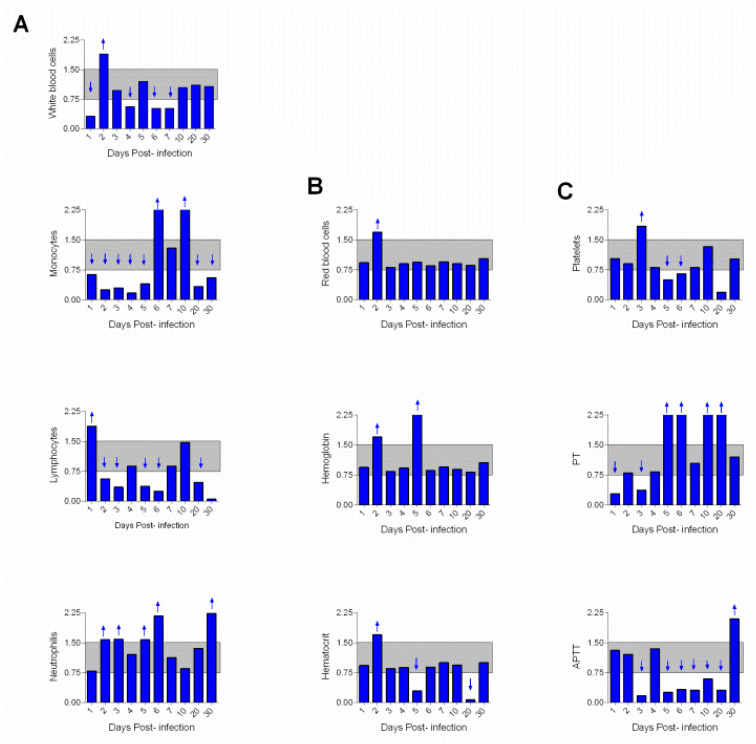
Hematological profile of squirrel monkeys (*Saimiri* spp.) infected with the yellow fever virus. (**A**) Leukogram, leukocytes, monocytes, lymphocytes, and neutrophils were measured. (**B**) Erythrogram, red blood cells, hemoglobin and hematocrit were evaluated. (**C**) Coagulogram, platelets, prothrombin time (PT), and activated partial thromboplastin time (TPPA) were verified. The data were transformed into a ratio where values were considered before and after infection. Normal values appear between 0.75 and 1.5 (gray band). The relevant differences are highlighted by the arrows in the figure (↓ = decrease < 0.75 and ↑ = increase > 1.5 in the baseline).

During infection, a marked decrease in leukocyte counts (1–7 dpi) was observed in the infected animals—monocytes (1–5 dpi) and lymphocytes (2–30 dpi)—as well as in neutrophilia at 2–6 dpi (Figure 2A). No relevant changes were observed in erythrogram, except for red blood cell enlargement at 2 dpi, decreased hemoglobin at 2 and 5 dpi, and hematocrit decrease at 5 and 20 dpi (Figure 2B). Thrombocytopenia was observed (5–20 dpi); and the PT was below normal values at 1–3 dpi and above normal between 5 and 20 dpi. The APTT decreased between 3 and 20 dpi (Figure 2C).

**Figure 3:**
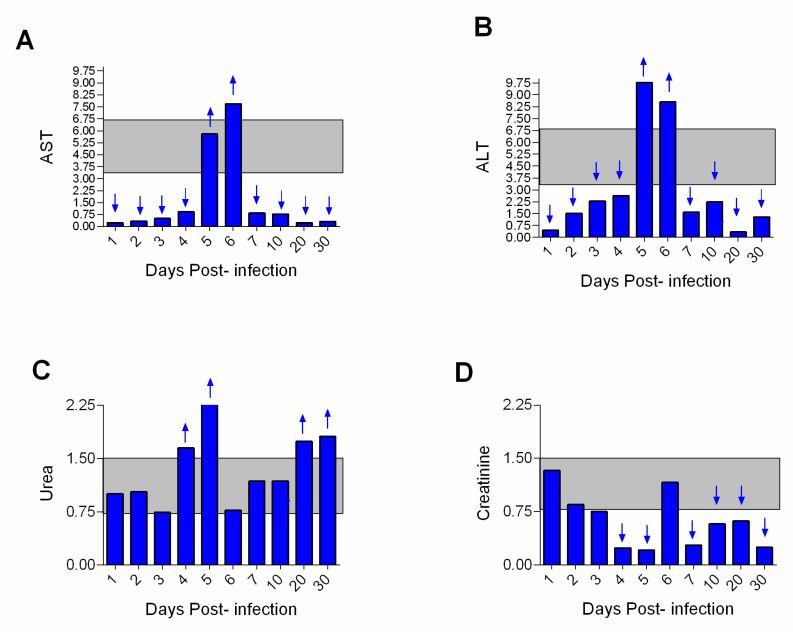
Biochemical tests of hepatic and renal function of squirrel monkeys (*Saimiri* spp.) infected with the yellow fever virus. (**A**,**B**) Relative ratio of the enzymes aspartate aminotransferase (AST) and alanine aminotransferase (ALT); (**C**,**D**) relative ratio of blood urea and creatinine. The data were transformed into a ratio where values were considered before and after infection. Normal values appear between 0.75 and 1.5 (gray band). The relevant differences are highlighted by the arrows in the figure ((↓ = decrease < 0.75 and ↑ = increase > 1.5 in the base).

Liver function showed an increase in AST at 5 and 6 dpi and ALT at 3–10 dpi. The highest levels of hepatic markers were found at 5 and 6 dpi. However, ALT values were higher than AST. In addition, AST was shown to be below normal (gray band) values between 1 and 3 dpi and 20 and 30 dpi (Figure 3A,B). Increased levels of blood urea were observed between 4 and 20 dpi and a marked decrease in creatinine occurred between 4 and 30 dpi (Figure 3A,B).

**Figure 4:**
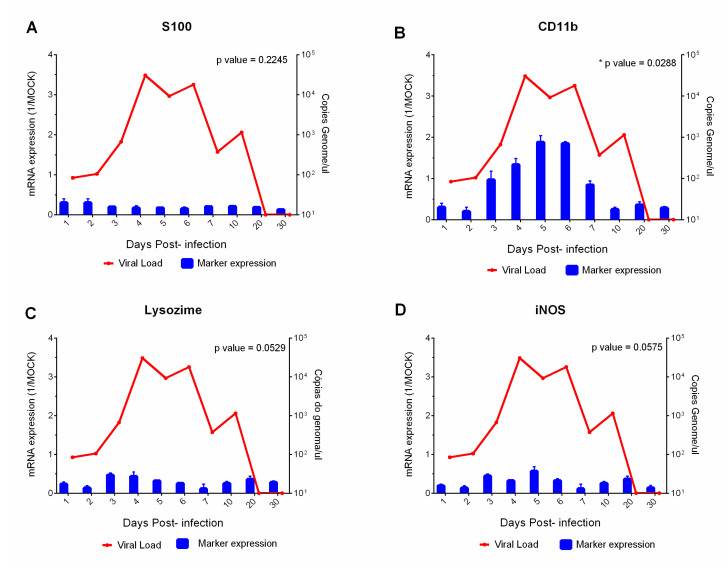
Correlation of viral load with serum profile of the cellular phenotype markers (S100 and CD11b) (**A**,**B**), stress and cell death (iNOS and lysozyme) (**C**,**D**), of squirrel monkeys (*Saimiri* spp.) infected with the yellow fever virus. Performing each sample in technical and biological triplicate increased the reliability of the generated data.

The S100 protein marker responsible for presenting viral antigens to helper lymphocytes was expressed at low levels throughout the experiment (1–30 dpi) (Figure 4A). The CD11b marker expressed on the leukocyte surface was detected throughout the experiment and its expression was directly proportional with YFV titers; it increased progressively with increasing of numbers of viral particles (3–7 dpi) and decreased between 20 and 30 dpi (Figure 4B). The detection of CD11b showed that these cells are directly associated with the M1 phenotype of macrophages, which was confirmed by the expression of lysozyme and iNOS (1–30 dpi), enzymes responsible for generating NO and ROS, which cause cell damage (Figure 4C,D).

**Figure 5:**
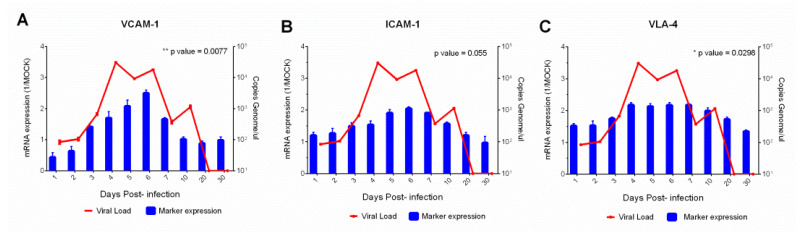
Correlation of viral load with serum profile of endothelial markers (VCAM-1, ICAM-1 and VLA-4) (**A**–**C**), in squirrel monkeys (*Saimiri* spp.) infected with yellow fever virus. Evaluating each sample in technical and biological triplicate increased the reliability of the generated data.

**Figure 6:**
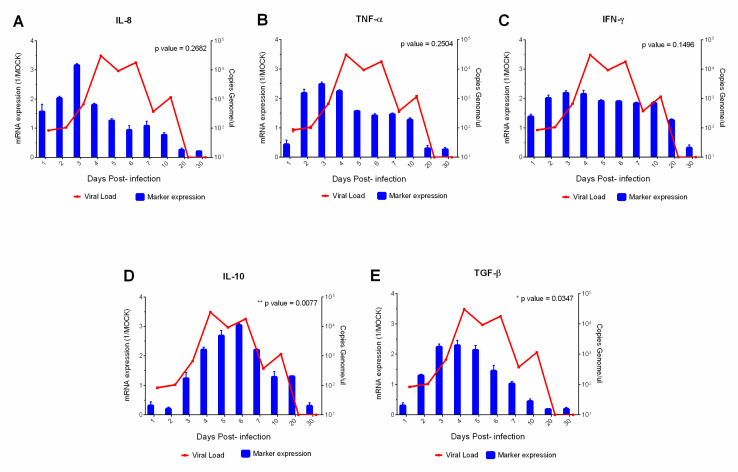
Correlation of viral load with serum profile of the proinflammatory (IL-8, TNF-α and INF-γ) (**A**–**C**) and anti-inflammatory cytokines (IL-10 and TGF-β) (**D**,**E**), in squirrel monkeys (*Saimiri* spp.) infected with yellow fever virus. Evaluating each sample in technical and biological triplicate increased the reliability of the generated data.

We analyzed the ICAM-1, VLA-4 and VCAM proteins in serum; these markers act on leukocytes binding to endothelial cells and assist in transmigration of leukocytes to inflammatory tissue. These endothelial cell markers were identified at high and constant levels throughout the study period (1–30 dpi) (Figure 5A–C). The highest levels of these serum chemokines coincided with the period of highest viral load (4–6 dpi). In addition, we also investigated the activation of the proinflammatory response which was evidenced by presence of IL-8 expressed at high levels in the initial infection phase (1–4 dpi) and gradually decreased at 5–30 dpi (Figure 6A).

TNF-α levels were similarly detected (Figure 6B); however, elevated levels lasted longer during the acute phase (1–7 dpi). IFN-γ levels (Figure 6C) were inversely proportional to the expression of IL-10 (Figure 6D). Meanwhile, high levels of circulating IFN-γ were expressed from 1st dpi and remained elevated between 1–10 dpi (Figure 6C); IL-10 levels increased between 1–6 dpi and decreased between 20–30 dpi (Figure 6D). High rates of IFN-γ and IL-10 coincided with peak viremia (4–6 dpi). In addition, TGF-β was expressed at progressive levels from 1 to 7 dpi and decreased over time (10–30 dpi) (Figure 6E). In summary, the levels of CD11b, IL-10, TGF-β, VCAM-1, ICAM-1 and VLA-4 showed a positive and significant correlation (*p* < 0.05) with viral load.

## 4. Discussion

The exquisite susceptibility of NHPs to YFV infection is well known; it was demonstrated almost a century ago when Stokes et al. [15] inoculated the prototype YFV isolate (Asibi strain) into rhesus monkeys and demonstrated that the virus could be transmitted from humans to NHPs. Several subsequent studies conducted by Davis [16,17] showed the susceptibility of South American NHP to YFV, although most animals showed no signs of disease. Davis found that animals of the genera *Callithrix, Leontocebus*, and *Saimiri* were susceptible to YFV infection and died within a few days of infection. However, the infection was irregular, and there was no hepatic injury similar to that described in the rhesus monkey livers infected with YFV. Similarly, our squirrel monkey model showed that infection occurred irregularly, and only one animal died at 6 dpi. However, our model showed high susceptibility to YFV doses similar to the amount of YFV found in the mosquito tissues and the infectious dose reportedly inoculated during vector bites [18].

The clinical data reported in our study illustrate in detail the different forms of presentation of the disease in squirrel monkey. The severe (fulminate) form exemplified that the infectious period was characterized by fever, chills, vomiting, diarrhea and prostration of squirrel monkeys ranging 3 to 4 dpi. The classic signs that characterize hepatic–renal failures—jaundice, albuminuria, anuria and hemorrhages—were also striking in the animal that died at 6 dpi, data published in Ferreira et al. [19]). Monath et al. [20] infected rhesus monkeys with a West African YFV genotype (Dak H 1729) isolated during the 1965 epidemic in Senegal and found that the Asian monkeys died between 4 and 6 dpi.

The most severe signs of disease observed in experimental animals coincided with the time at which viral genome was detected (1–10 dpi). Approximately 19 × 10^3^ genome copies/μL of the YFV were detected in the animal with a fatal outcome at 6 dpi, a lower viremia when compared with the viremic peak (30 × 10^3^ genome copies/μL) detected in the animal euthanized at 4 dpi. Engelmann et al. [21] infected several groups of rhesus monkeys with different infectious doses of YFV isolated from a human with severe disease. Infectious viral titers ranged from 25 to 5 × 10^4^ TCID50. Infection with the maximum dose (5 × 10^4^ TCID50 of YFV) resulted in a fulminant disease lasting on average 4–7 days. The viremia peak occurred between 3 and 7 dpi, and titers reached 10^9^ to 10^13^ viral copies/mL. Interestingly, two animals infected with the lowest dose of YFV (25 or 100 TCID50) had two rounds of viremia, one between 3 and 5 dpi and another between 10 and 14 dpi. Although our animals did not show any viremia peaks as high as those obtained by those authors, the infectious dose was able to produce viremia and clinical signs in the experimental animals. Further studies are necessary to determine whether infection with higher or lower doses may or may not result in two periods of viremia and follow a timeline similar to that seen in humans.

Activation of the immune response was marked by the presence of IgM-specific and total anti-YFV antibodies (5–30 dpi). The surviving infected animals showed signs of mild disease only in the first days of post YFV infection (1–9 dpi). In humans, specific anti-YFV IgM antibody levels rapidly increase after 6 days from disease onset and remain detectable for up to 3 months by the IgM- ELISA. Total antibody detection in our study was early, with high HI titers detected at 6 dpi (1:640) and 7 dpi (1:1280), which declined at 30 dpi (1:80). In humans, the peak antibody titers usually occur between 30 and 60 days, and then slowly decline after 6 months [22]. The detection of cross-reactivity with YFV (vaccinal strain—17D) and other flaviviruses (DENV 1-4, ILHV, SLEV and ROCV) between 5 and 30 dpi, as shown in our study, resemble those described by several other studies demonstrating the intense cross-reactivity of YFV with other flavivirus in NHP and humans [23].

The most relevant finding in the erythrogram of the squirrel monkeys was the decrease in hematocrit observed between 5 and 20 dpi. Conversely, the leukocytes presented significant changes, with leukopenia between 1 and 7 dpi coinciding with the viremia peak detected between 4 and 6 dpi. Decreased counts of monocytes (1–5 dpi) and lymphocytes (2–30 dpi) as well as neutrophilia (2–6 dpi) were also observed. In humans, leukopenia with neutropenia and lymphocytosis is frequent. As the disease progresses, leukopenia may increase, and in this case, the leukogram exhibited values between 1000 and 2000 leukocytes/mL of blood [24].

Studies conducted by Engelmann et al. [21] have shown that rhesus monkeys experimentally infected with YFV showed a slight decrease in total leukocyte count between 4 and 6 dpi. This leukopenia was more evident in the animals with highest viral loads. This contrasts with that observed in humans with the disease, where thrombocytopenia can reach values ≤50,000 platelets/mm^3^. The main findings of our study were values above the normality of PT between 5 and 20 dpi and discrete thrombocytopenia (5–20 dpi). APTT decreased between 3 and 20 dpi in contrast to findings in human studies [25].

Liver enzyme markers were shown to be increased with peak of AST at 5 and 6 dpi and of ALT at 3–10 dpi. The highest levels of these enzymes were found at 5 and 6 dpi, which also coincides with the viremia peak and the intense hepatic impairment of the infected animal that died at 6 dpi. In humans, increased aminotransferases levels are observed 2–3 days after the onset of symptoms and peak 5–6 days, as well as AST values exceed ALT values and is a poor prognostic sign. The findings of this study were different (ALT > AST), presumably due to the cytopathic effect of the virus on myocardium and skeletal muscles. It has also been shown that in humans, AST values greater than 1000 U/L are indicative of disease associated with extensive hepatic tissue injury, as usually occurs in severe cases [26], and they may remain elevated for a period of up to two months in convalescent patients.

Tests of renal function have shown increased levels of urea (4–20 dpi). In humans, urea values can reach up to 5 or 6 times the normal values, or even higher, in YF infections. The creatinine levels were irregular and below normal (4–30 dpi). In humans with YF, creatinine values above 2.0 mg/dL are usually observed, and may be indicative of renal complications [25]. This intriguing result remains to be explained.

The in situ immune response to YFV infection is sparse and paradoxical to the intense tissue injury [27], in contrast to that observed with DENV, in which the immune response produced during infection is closely associated with the severity of the case [28]. In humans, the organic dysfunctions are directly caused by the YFV or due to secondary immunological reactions to the infection. The major components of the immune responses to YFV infection include TNF-α, TGF-β and IFN-γ and these (especially TGF-β) are related to apoptosis and the various symptoms or signs observed in severe disease such as vascular leak syndrome, thrombocytopenia, aminotransferase abnormalities, vomiting and hemorrhagic diathesis [29].

In our experimental study, all Th1 response markers (TNF-α, INF-γ, INF-β); Th2 (TGF-β, IL-10, IL-4), endothelial markers (VCAM-1, ICAM-1, VLA-4); S100, CD11b, IL-8, lysozyme and iNOS were expressed irregularly and followed the dynamics of the period in which the viral genome was detected. The expression of CD11b showed a direct association with the M1 phenotype. This is confirmed mainly by the expression of lysozyme and iNOS, enzymes responsible for generating NO and ROS that cause cellular damage. The deleterious effects and antiviral response are dependent on the specific response of M1 macrophages and cytokines that activate these cells, such as TNF-α, IFN-α and IFN-γ. There was no antagonistic action of Th1 profiles on Th2 and vice versa, which may have favored viral clearance, homeostatic control, and the survival of most animals.

There are few studies describing the immune response in the peripheral blood of NHPs and other experimental models. In one rhesus study [21], the levels of IL-4, IL-5 and IL-8 were unchanged or below levels considered as basal. In contrast, increased levels of IL-6, MCP-1, IL-15 and IFN were detected, mainly shortly before euthanasia, and the levels of each cytokine showed a significant correlation with viral load [21]. The limited number of animals is considered a limitation of the study but is justified by the rational use of animals in experimentation established by the ethics committee. We emphasize that the data must be analyzed with caution.

## 5. Conclusions

Collectively, the results obtained in this study with squirrel monkeys showed that those NHPs are a good model for studies on immunopathology of YFV infection, due to several findings including clinical changes similar to those observed in humans.

## Data Availability

Data can be requested from the corresponding author.

## Supplementary Materials

The following supporting information can be downloaded at: https://www.mdpi.com/article/10.3390/v15030613/s1, Table S1: Initiators designed using the Primer 3 Plus program, Table S2: Detection of IgM/IgG antibodies to viruses belonging to the *Flavivirus* genus in non-human primates (*Saimiri* spp.) infected with the yellow fever virus.
