# Supplementary material for: Experimental Yellow Fever in the Squirrel Monkey (Saimiri spp.): Hematological, Biochemical, and Immunological Findings
Source: Viruses. 2023 Feb 23;15(3):613. doi: 10.3390/v15030613 (PMC10052740; doi:10.3390/v15030613)
Supplement: Supplementary file 1 [file viruses-15-00613-s001.zip › viruses-1937104-supplementary.pdf]

Table S1. Initiators designed using the Primer 3 Plus program

	**Sequencie 5´-3´**
YFV	F -GCTAATTGAGGTGYATTGGTCTGC
	R -CTGCTAATCGCTCAAMGAACG
S100	F – ATAAGAGGTTGCCCCTGTGC
	R – TCCTTGTCTCCTTGCTGCAG
CD11b	F – AGCCTGGTATTCTTGGTGCC
	R – AGCCAGAAAGTCGGTGTGAG
iNOS	F – GCTGGAGGAGTTTCTGTCCC
	R – TTCCAGATCCTCCTCCCTGG
lysozyme	F – CGGTCCAGGGCAAGATCTTT
	R – CACCCAGTTTGCTAGGCTGA
VCAM-1	F- TAAAATGCCTGGGAAGATGG
	R- CACAGGCTGCGAGTCCCCAT
ICAM-1	F – CAAACCTTTGACCTGCCAGC
	R – GGAGACCTGAGCCTCTGAGA
VLA-4	F – TTAATTGTGATGGGAGCCCC
	R - AGACAAAAAGAGAGCCAGTCCA
IL-8	F- GCTGGCGGTGGCTCT
	R- CAAATTTATCAAAGAAC
IFN-γ	F- CTTGGCTTTTCAGCTCTGCG
	R- CTGTCACCCTCCTCTCTCCA
TNF-α	F - GGAGCTGGCCGAGGA
	R - AAGGACGAGCTCTCC
IL-10	F- AGCCTTGTCTGAGATGATCCAGTTT
	R- GACGCCTTTCTCTTGGAGCTTACTA
TGF-β	F- CGTGCGGCAGCTGTATATTG
	R- GTACTGTGTGTCCAGGCTCC

Table S2. Detection of IgM/IgG antibodies to viruses belonging to the *Flavivirus* genus in non-human primates (*Saimiri spp.*) infected with the yellow fever Virus.

**dpi**	**Virus**								
	**YFV**	**YFV-17D**	**ILHV**	**ROCV**	**SLEV**	**DENV-1**	**DENV-2**	**DENV-3**	**DENV-4**
1	-	-	-	-	-	-	-	-	-
2	-	-	-	-	-	-	-	-	-
3	-	-	-	-	-	-	-	-	-
4	-	-	-	-	-	-	-	-	-
5	1:40	-	1:20	-	-	-	-	-	-
6	1:320	1:160	1:160	-	-	-	1:160	-	1:20
7	≥1:640	≥1:640	1:320	1:40	1:160	1:320	1:160	-	1:160
10	1:160	≥1:640	1:320	1:20	1:160	1:320	1:160	1:160	1:160
20	1:160	≥1:640	1:320	1:20	1:160	1:320	1:160	1:160	1:320
30	1:80	1:80	-	-	-	-	-	-	-

YFV = *Yellow fever Virus* (wild and vaccinal strain - 17D); DENV 1, 2, 3, 4 = *dengue Virus* 1-4; ILHV = *Ilheus Virus*; SLEV = *Saint Louis Encephalitis Virus*; ROCV = *Rocio Virus*; dpi = Days post infection; (-) negative.
